# Dynamic Resource Allocation and Forecast of Snow Tourism Demand Based on Multiobjective Optimization Algorithm

**DOI:** 10.1155/2022/4606289

**Published:** 2022-06-03

**Authors:** Mingfen Feng, Xiaomei Zhang, Shuqi Dong

**Affiliations:** ^1^College of Economics and Management, Northeast Agricultural University, Harbin 150038, China; ^2^Department of Economics, Heilongjiang University of Finance and Economics, Harbin 150025, China; ^3^Wenzhou Business College, Wenzhou 325035, China

## Abstract

This paper aims to map the task of reliable transmission of wireless sensor networks. At the same time, this paper transforms the mapping problem of wireless sensor networks into a problem of reducing the energy consumption of mapping under many constraints such as reliability and scheduling length and uses discrete particle swarm optimization algorithm to map. For optimization, the algorithm performs iterative calculations to obtain the best mapping node for each operation so that the inertia coefficient of the existing particle swarm optimization algorithm is improved and linearly minimized with the number of iterations. When resource-demanding tasks need to allocate dynamic resources to multiple nodes to complete collaboratively, adding the mapping principle of the nearest node in the discrete particle swarm optimization mapping reduces the energy consumption of communication between tasks. An in-depth analysis of the influencing factors of the ice and snow tourism market shows that the per capita disposable income of urban residents and the number of urban residents have a significant impact on the ice and snow tourism market demand. In addition, regression analysis and demand-based forecasting are important methods to analyze the scale and development trend of tourism. At the same time, it shows an important position in the purpose of urban tourism and regional market share so as to provide a basis for decision-making in tourism destination marketing. This paper mainly studies and analyzes the wireless sensor network and further introduces it into dynamic resource allocation and ice and snow tourism, which can promote the continuous development of dynamic resource allocation and ice and snow tourism.

## 1. Introduction

To create credible transmission, this article mainly uses the multipath transmission mode to optimize the repeater and send data to the node. In the wireless sensor network relay area, select the largest and suboptimal node in the wireless sensor network relay area as the next hop node of the relay, and the relay is then selected to obtain multiple transmission paths [1]. And in order to improve energy efficiency and prolong the life of the network, we studied the design of dynamic resource allocation for the Multi-Radio Wireless Sensor Network (MRWSN) based on the multiradio frequency technology to allocate the maximum power and channel interface in each radio frequency in the network and establish deployment nodes [2]. Energy efficiency model combines the energy efficiency of the node and the remaining energy factor at the same time. The energy allocation model is a distributed resource optimization game model, and MRWSN has designed and confirmed an efficient game plan in the homeopathic game based on the energy allocated resources, and the algorithm can converge to the Nash equilibrium [3]. Experimental results show that the algorithm not only can effectively reduce energy consumption but also can significantly improve the energy efficiency and capacity of the network, and it is also of great significance for ice and snow tourism analysis [4]. This paper analyzes the positive correlation between urban residents' per capita disposable income, urban population, and ice and snow tourism market demand; draws a conclusion that is consistent with the analysis of the factors affecting ice and snow tourism market demand; and establishes a revised estimation model for urban tourism planning and development [5]. And useful suggestions are provided for scientific judgment. This study draws on the experience of domestic and foreign ice and snow tourism service system construction and interviews relevant experts based on specific conditions [6]. It is believed that the elements of the ice and snow sports tourism service system are the management system, the product system, and the service system of the management system [7]. The construction of the ice and snow tourism service system should have the functions of guidance, evaluation and management, including the management system, supply system, commodity system, and ice and snow tourism service system [8]. The management system is divided into four levels: government administrative departments, sports bureaus, tourism development committees and other administrative units, and ice and snow sports tourism industry associations; the supply system includes leisure and entertainment venues, tourism companies, public welfare clubs, and lucrative sports companies; The commodity system includes ice and snow tourism products, spring leisure tourism products, winter festival tourism products, and national traditional ice and snow tourism products; and the service system includes supporting equipment and facilities, auxiliary equipment items, and services [9]. By dividing these systems and forecasting the demand of the ice and snow tourism market, it will help to improve the local ice and snow tourism service industry.

## 2. Related Work

The literature introduces the configuration structure, application fields, characteristics, and composition structure of WSN wireless sensor nodes, and then, we clarify the research background and importance of WSN and study the important role of power and channel sources in optimizing network performance. Finally, we summarize the research status of resource allocation algorithms in WSN and point out the advantages and disadvantages of existing algorithms, which provides a basis for the research of this article [10]. The literature introduces the influence and nature of power control and channel allocation on network formation, establishes the energy consumption model and node error rate model, optimizes multipurpose distributed resource allocation, links disconnection protection conditions, links capacity and network connectivity models, and then can derive energy consumption and bit error rate. Finally, Pareto's theoretical analysis result is the best solution to establish the model [11]. The literature introduces a distributed resource allocation model and designs a multipurpose optimization algorithm for distributed resource allocation based on particle allocation to obtain the best resource allocation plan. The algorithm is mainly divided into two steps. In the initial stage, the adjacency table is obtained, and the maximum power and flow are selected in the multipurpose particle swarm optimization stage [12]. The theoretical analysis can confirm the time complexity and information complexity of the algorithm. Finally, the feasibility of the algorithm is proved by the simulation experiment and analysis of the generation algorithm. The literature introduces an efficient distributed resource allocation algorithm that allows network nodes to easily join the Nash equilibrium and maximize node benefits [13]. After that, through theoretical analysis such as homeopathic game, we proved that the given origin allocation algorithm can converge to the Nash equilibrium and use the Pareto optimal theory to evaluate the effectiveness of the game. Finally, simulate the MATLAB experiment to analyze the influence of the proposed game algorithm on network generation. The literature introduces a reliable and efficient task mapping based on discrete particle swarm optimization and then studies the optimal node assignment for task mapping [14]. First, the task mapping is introduced and the task mapping is created for the preparation work. Then, the task mapping is converted into the optimal solution problem of the task mapping to the node energy consumption under multiple constraints, and the mathematical model is established. From the number of iterations, we use the dynamic weight factor that decreases linearly with the number of iterations to obtain the model node with the least energy consumption, and then, we use the near-node principle to map the resource-heavy tasks required for multiple nodes to work together and finally complete the mapping operating [15].

## 3. Research on Optimization Algorithm for Distributed Resource Allocation in Wireless Sensor Networks

### 3.1. Basic Architecture of Wireless Sensor Network

Wireless sensor network is a large-scale physical network composed of multiple sensor nodes. Nowadays, wireless sensor network has been widely used by researchers all over the world.

**Figure 1:**
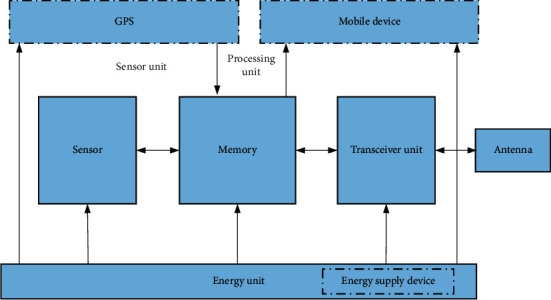
Hardware architecture of sensor node.

The sensor node is a small embedded device with low price and low power consumption, but it also has the disadvantages of weak processor processing power and low memory storage capacity. The sensor node performs simple initial processing on the acquired data, forwards it to the next hop node of the base station, and transmits the data of the base station to the end user through the Internet, satellite, etc. A wireless sensor device (node) is mainly composed of four main components. Its structure diagram is shown in Figure 1. The communication device is responsible for preventing the exchange of data and information with other nodes, and the energy supply provides the necessary energy for the node of the sensor network.

Wireless sensor network is a self-organizing network system composed of sensor nodes with data processing and wireless communication capabilities. In addition to this, the network collects information about objects that are perceived in the covered geographic area, processes it, and then sends it to the recipient.

#### 3.1.1. Energy Model of Wireless Sensor Network

**Figure 2:**
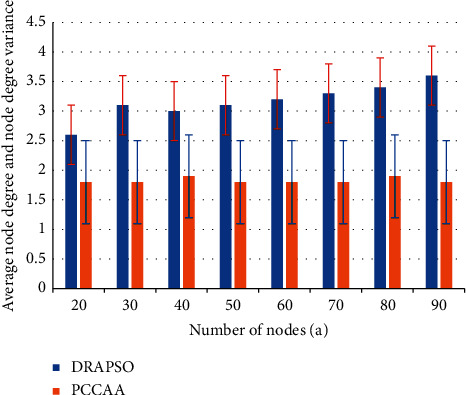
Comparison of average node degree and node degree variance.

The energy model used in this chapter is a simplified wireless communication power model, and its structure is shown in Figure 2. The energy model is the energy consumption when the task is transmitted on the node. The energy consumption mainly includes the energy consumed by the sending task, the energy consumed by the receiving task, and the energy consumed by the power amplifier when the node sends the task (negligible).

Figure 2 Energy model of wireless sensor network.

Consistent with the wireless sensor network energy model, the formula is

(1) $$\begin{array}{c} E_{i}=E_{T}+E_{R}. \end{array}$$

Among them, the energy consumed by data transmission is recorded as ET, which mainly combines the energy lost during the data transmission distance *d*.

(2) $$\begin{array}{c} E_{T}=E_{\text{elec}}+\alpha d^{\tau }. \end{array}$$

Since the ER energy received by the node is the standard *E*_elec_ of data, the total energy consumption of node *i* can be expressed as

(3) $$\begin{array}{c} E_{i}=E_{\text{elec}}+\alpha d^{\tau }+E_{\text{elec}}. \end{array}$$

After the tasks are mapped to the sensor nodes, most of them are selected based on the centrality and strength of the nodes in the area. At the same time, the two suboptimal nodes send their work to the next hop node, and then, the relay node is based on the two nodes. The best child node to select the relay until the work is transferred from the source node to the destination node, forming multiple transmission paths.

### 3.2. Multiobjective Optimization Algorithm Design for Distributed Resource Allocation Tasks

#### 3.2.1. Task Mapping Problem Description

The main purpose of task management is to reduce the energy consumption of task mapping under several constraints such as the reliability of task mapping. This article believes that the communication between the control center node and the sensor node (that is, within the node) is completed in a faster way. So, this communication may not matter. Given a set of tasks and a set of mapping candidates, a suitable node can be found for the task and the task can be executed with minimal energy consumption under reliability constraints, communication relationships, and deadlines. In this way, it is best to map the task on the node. The NP problem becomes a very complex multiconstraint solving problem, which mainly includes the following three constraints:

(1) If the reliability of the communication link between nodes can be guaranteed, it provides a guarantee for reliable transmission of work.
  (4) $$\begin{array}{c} \text{min}E_{\text{map}}=w_{1}\cdot \text{min}\left(c\left(i\right)\right)+w_{2}\cdot \text{min}\left(E\left(n\right)\right). \end{array}$$
(2) *Restriction of communication relationship*: when the direct predecessor of a job is assigned to another node, the processing activity can only be started after receiving the processing result of the direct tracking job, and the control is limited to the job schedule.
  (5) $$\begin{array}{c} \text{s.t }X_{t}^{m,n}\in \left\{0,1\right\},\forall n\in N,\forall m\in M,\forall t\in T. \end{array}$$
(3) Due to the deadline, the work processing must be completed before the deadline. The mathematical model for creating the mapping node operation is

(6) $$\begin{array}{c} \sum_{t\in T}\sum_{n\in N}X_{t}^{m,n}=1,\\ \sum_{m\in M}Y_{t}^{m,n}\leq 1,\\ \text{lengt }n\left(H\right)\leq DL\;,\\ R_{\text{map}}\geq \varepsilon . \end{array}$$

The time to perform *m* operations on *k* nodes can be expressed as ∑_*i*=1_^*m*^*T*_*i*_, , where *T*_*i*_ represents the time to perform the *i*th operation. Therefore, the reliability of node *k* can be expressed as

(7) $$\begin{array}{c} R_{k}=e^{-\lambda_{k}\sum_{i=1}^{m}T_{i}},\forall i\in m,\forall k\in n. \end{array}$$

Similarly, the reliability of the communication link can be compared with the reliability of the node. If tasks *i* and *j* are mapped to nodes *k* and *s* at the same time, the reliability of the communication link is the one between the nodes mapped to tasks *i* and *j*. Path_*ij*_ between tasks is satisfied by the reliability of E in the communication between tasks.

(8) $$\begin{array}{c} R_{{\text{path}}_{ij}}=e^{-\mu_{ks}\sum_{e_{ks}\in {\text{path}}_{ij}}tc_{ij}/p_{ks}},\forall i,j\in m,\forall k,s\in n. \end{array}$$

After that, the overall reliability of task mapping is

(9) $$\begin{array}{c} R_{\text{map}}=\prod_{k=1}^{n}e^{-\lambda_{k}\sum_{i=1}^{m}T_{i}}\prod_{\forall e_{ks}\in {\text{path}}_{\tilde{j}}}e^{-\mu_{ks}e_{k_{s}}\sum_{{\text{path}}_{ij}}tc_{ij}/p_{ks}},\forall i,j\in m,\forall k,s\in n. \end{array}$$

#### 3.2.2. Establishment of Energy Efficiency Model

The optimized relay transmission distance is determined by the number of hops that the node energy can transmit, *n*_*op*_. At the same time, it takes the least energy from most hops. The specific derivation process is as follows:

(10) $$\begin{array}{c} C_{i}=\sum_{i=1}^{h}E_{T}+\sum_{i=1}^{h-1}E_{R},\\ =\sum_{i=1}^{h}\left[E_{\text{elec}}+\alpha {\left(x_{j}-x_{i}\right)}^{\tau }\right]+\sum_{i=1}^{h-1}E_{\text{elec}}. \end{array}$$

In order to reduce energy consumption, the average inequality is used to obtain the minimum energy consumption. The distance *d*(*k*,*s*) from the *k*th node to the sink node *S* is

(11) $$\begin{array}{c} d\left(k,s\right)=S-x_{k}=\sum_{i=1}^{h}\left(x_{j}-x_{i}\right),\\ C_{i}\geq \left(2h-1\right)E_{\text{elec}}+\frac{\alpha {\left(S-x_{k}\right)}^{\tau }}{h^{\tau -1}}. \end{array}$$

And so,

(12) $$\begin{array}{c} C_{i}^{\text{min}}\left(h\right)=\left(2h-1\right)E_{\text{elec}}+\frac{\alpha {\left(S-x_{k}\right)}^{\tau }}{h^{\tau -1}}. \end{array}$$

Then, we get the source of the hop count based on the minimum energy consumption.

(13) $$\begin{array}{c} \frac{\partial C_{i}^{\text{min}}}{\partial h}=2E_{\text{elec}}-\left(\tau -1\right)\frac{\alpha {\left(S-x_{k}\right)}^{\tau }}{h^{\tau }}=0. \end{array}$$

Get

(14) $$\begin{array}{c} h_{op}=\frac{{\left[\alpha \left(\tau -1\right)\right]}^{1/\tau }\left(S-x_{k}\right)}{{\left(2E_{\text{elec}}\right)}^{1/\tau }},\\ {\text{d}}_{op}=\frac{S-x_{k}}{h_{op}}=\frac{{\left(2E_{\text{elec}}\right)}^{1/\tau }}{{\left[\alpha \left(\tau -1\right)\right]}^{1/\tau }},\;d_{op}<R_{c}. \end{array}$$

Among them, *Ci* represents the comprehensive strength of the *i*th node, and the maximum number of hops for data transmission is represented by *h*_*op*_. This chapter selects through energy consumption and calculates the optimal number of hops for data transmission according to the energy in the transmission process.

#### 3.2.3. Multiobjective Particle Swarm Optimization Algorithm

Each sensor in WSN uses information exchange to generate its own neighbor list list(*i*). The header format of the sorted list is shown in Table 1, where the set of child nodes *i* is designated as child node child(*i*).

In this article, we use the MOPSO method to solve the problem of optimizing the combination of power and channel resources, taking into account the mutual influence between energy consumption and BER and obtaining a trade-off between different goals. In WSN, each node uses an ID to perform multiple optimization purposes. When a node in the network updates the multipurpose optimization amount, the power and channel state of other nodes will not change.

Any solution can be expressed as an *X*_*i*_ matrix with two rows and two columns of *L*, where two rows and two columns of *L* represent power and channel allocation, so a single code can be expressed as

(15) $$\begin{array}{c} X_{i}\left(m\right)=\left[\begin{array}{c} x_{1,1},x_{1,2}\ldots x_{1,L-1},x_{1,L}\\ y_{2,1},y_{2,2}\ldots y_{2,L-1},y_{2,L} \end{array}\right]. \end{array}$$

MOPSO starts by randomly initializing the velocity and position of the particles and iteratively finds the most effective solution. In the *k*th iteration, each particle is oriented by two integer values, and in other words, the solution obtained is changed. These two extreme values make *P*best_*i*_^*k*^ the best position that all groups will mark and experience the best position in *G*best^*k*^. The particle velocity and position are updated as shown in equations (16) and (17).

(16) $$\begin{array}{c} V_{i}^{k+1}=w\cdot V_{i}^{k}+c_{1}\cdot r_{1}\left(P{\text{best}}_{i}^{k}-X_{i}^{k}\right)+c_{2}\cdot r_{2}\left(G{\text{best}}^{k}-X_{i}^{k}\right), \end{array}$$

(17) $$\begin{array}{c} X_{i}^{k+1}=X_{i}^{k}+V_{i}^{k}\;k\in \left(1,2,\ldots ,K\right). \end{array}$$

The result of the multipurpose problem of optimizing distributed source allocation is the Pareto solution set. Because of the final requirements of WSN in practical applications, it is necessary to select a suitable solution as the final application design according to the specific environment and performance, so it needs to be found in EA the best solution. This component uses fuzzy decision-making technology to derive the final optimization plan, allocates resources from the poor solution, first corrects the two indicators of BER performance and energy consumption, and obtains the linear membership of each performance indicator *f*_*i*_ as follows:

(18) $$\begin{array}{c} u_{i}=\frac{f_{i}-f_{i}^{\text{min}}}{f_{i}^{\text{max}}-f_{i}^{\text{min}}}. \end{array}$$

Equation (18) shows that the number of membership functions is positively correlated with the level of awareness of behavioral goals. For each nonvulnerable EA solution, the aggregation function can be expressed as

(19) $$\begin{array}{c} P^{x}=\sum_{i=1}^{m}u_{i}^{x}. \end{array}$$

The boundary mapping node *θ* represents the ratio of the task resource requirement in the wireless sensor network to the resource provided by the node, which can be expressed as

(20) $$\begin{array}{c} \theta =\frac{\text{max}/m_{i}\in MC^{*}\left(m_{i}\right)}{\text{max}/n_{j}\in Ng\left(n_{j}\right)}. \end{array}$$

### 3.3. Simulation Experiment and Performance Analysis

#### 3.3.1. Simulation Parameter Setting

In this section, simulation experiments and performance evaluation of the DRAPSO algorithm are carried out in the MATLAB environment. In addition, the PCCAA algorithm comprehensively considers the node power, optimizes the combination of channel allocation, and makes the best selection of the power and channel of all nodes. The later results show that the algorithm can effectively reduce network failures and energy consumption. Therefore, this section compares the PCCAA algorithm with the DRAPSO algorithm and then specifically examines the performance of the PCCAA algorithm and the DRAPSO algorithm to verify the feasibility and effectiveness of the given algorithm. The experimental simulation area of WSN is 200 m× 200 m square, the experimental parameters of the two algorithms are the same, and other parameter settings are shown in Table 2.

#### 3.3.2. Comparison of Average Node Degree and Node Degree Variance

**Figure 3:**
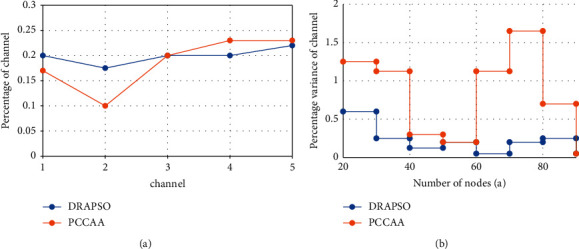
Comparison of channel equalization. (a) Percentage of channel; (b) percentage variance of channel.

We randomly assign 20 to 90 nodes in a 200 m × 200 m grid with a channel number of 5 and then use the PCCAA and DRAPSO algorithms to obtain a comparison graph comparing the average node degree and the node degree variance of the network. The result is shown in Figure 3. The average node degree in the network represents the density of communication links and the degree of damage resistance of the network. It can be seen from Figure 2 that the average node level of the DRAPSO algorithm is much greater than that of the PCCAA algorithm. This is because the DRAPSO algorithm comprehensively considers the creation of the network when constructing the topology. Therefore, if there is a lot of external interference in the network and some nodes fail, the DRAPSO algorithm with a higher average node level can communicate normally without losing the network connection, which can extend the life of the network.

In the process of constructing a WSN topology, the smaller the node degree difference, the smaller the node degree difference between all nodes in the network, the closer they are, and the more similar the constructed topology. The length of the red and blue lines indicates the size of the difference in the degree of nodes in the network. From the results of the comparison chart, it can be seen that the DRAPSO algorithm has less node degree fluctuations, the network link distribution is more balanced, and the topology is better than the PCCAA comparison algorithm.

#### 3.3.3. Comparison of Channel Equalization

In the WSN channel allocation process, the balance size is taken as one of the standards. Through this standard, the measurement of channel network allocation becomes very important. At the same time, the imbalance of channel allocation will cause many links or nodes to use the same channel for data transmission at the same time. In this case, each node is disconnected from each other and contradicts each other, which affects and interrupts the entire network, causing more energy consumption and data loss. Therefore, in order to achieve the balanced use of spectrum resources, it is necessary to increase the balanced channel utilization rate and enhance the network resistance strength. The comparison result of the channel equalization algorithm and DRAPSO PCCAA is shown in Figure 3.

During the experimental phase, set the number of network nodes to 30 and the number of available channels to 5, then the channel diagram of the DRAPSO and PCCAA algorithms is shown in Figure 3(a). In Figure 3(a), it can be seen that the channel ratio of the DRAPSO algorithm is not much different, it is relatively flat, and the channel allocation is balanced. The difference in channel percentage represents the balance of network channel allocation. As channel 5 and the number of nodes increase from 20 to 90, the difference in the recorded network channel percentage is specifically reflected in Figure 3(b). It can be seen from the figure that the DRAPSO algorithm is in channel allocation. In the process, it has a smaller channel ratio than the PCCAA algorithm. The experimental results show that DRAPSO has more balanced channel allocation to the network topology and more efficient spectrum resources.

#### 3.3.4. Comparison of Network Average Power

**Figure 4:**
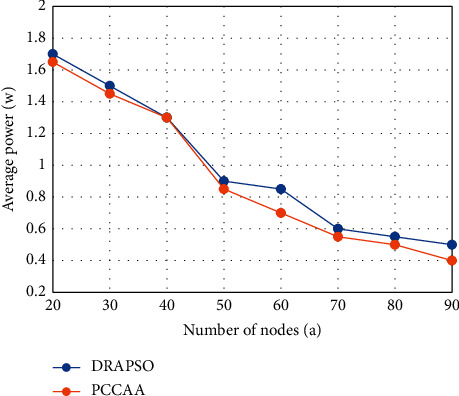
Average power comparison chart.

In the simulation step of this experiment, we set the number of channels to 5. If the number of nodes in the network is changed from 20 to 90, we can get the average power of the entire network. The final result is shown in Figure 4.

In WSN, the average network strength directly affects network performance. If the average power is too large, the node will consume more energy and shorten its lifespan. The lower the average network strength, the lower the network capacity, and the network data loss may be much more serious. Therefore, proper transmission power can effectively improve the quality factor of the network. Because the DRAPSO algorithm comprehensively studies the network energy consumption target and BER, taking into account network failures and network strength, the average power of the DRAPSO algorithm is slightly greater than that of the PCCAA algorithm. And because the PCCAA algorithm allocates power and channels to nodes based on their remaining energy, power gain is the minimum power that maximizes the benefit function under the condition of ensuring network connectivity. Therefore, it can be considered that the DRAPSO algorithm can reduce energy consumption and BER while ensuring network performance, thereby increasing network strength.

## 4. Research on Tourism Demand Forecasting Model Based on Multisource Data

### 4.1. Analysis of Influencing Factors of Ice and Snow Tourism Market Demand

Tourism demand is a special kind of consumption, and the overall demand is constantly changing. The factors that restrict and affect the changes in tourism market demand are complex, usually including tourists' origin, destination, tourists themselves, and many other factors. From an economic point of view, the economic status of residents is an important condition that affects tourism development. Residents first consider their own economic status when traveling. Therefore, the main economic factor that determines the market demand for ice and snow tourism is whether there are enough potential tourist sources. There is a correlation between the actual discretionary income and the size of tourism desire. Under the circumstance that other factors remain unchanged, the more people's discretionary income, the increase in the number of tourists, and the number of trips, especially when the number of people is increasing, the per capita GDP and per capita disposable income will also increase at a high speed. Ice and snow travel provides a useful basis.

Demographic analysis is an important standard and basis for market analysis. The size of the population determines the overall market demand and can form a large-scale tourism market, which must be based on a certain number of people. China is the most populous country in the world. The huge population size and continuous growth trend have created favorable conditions for increasing the growth of the city's ice and snow tourism market, which can become the foundation and driving force for the continuous growth of the ice and snow tourism market. From the perspective of the overall composition of the tourist ice and snow field, domestic tourists accounted for 96%, and foreign tourists accounted for only 4%. Therefore, the research on ice and snow tourism market demand mainly takes the number of domestic tourists as the main indicator of demand analysis, establishes many relationships with related influencing factors, and then makes more scientific predictions about future trends.

### 4.2. Design of Ice and Snow Tourism Service System Based on Reliability Prediction

This system is a tourism service system that integrates many network services. The data layer is a system database that stores system-related data information, including travel service information, user information, and related performance data collected by the system when users call travel services. The technical solution layer mainly includes four modules: service invocation, reliability prediction, service category selection, and service combination. When a travel service is called, it needs to collect data related to its performance, and these data can be used to predict reliability. After the reliability prediction, the prediction results can be provided to single-type service recommendation and service combination as the basis for selecting travel services. In other words, it calls the remote server Web service, and the user puts forward specific requirements to the interface calling layer based on the results provided by the technical solution layer, and the interface calling layer requests the network to access the service, then accepts the request, and returns the corresponding result.

The system uses relational MYSQL database for data storage, and according to different needs, a total of 9 database tables are designed.

1. *User information table*: as shown in Table 3, relevant basic user information is stored
2. *Travel service information table* (travel_service): as shown in Table 4, basic information related to travel services is stored
3. *Service category (category) table*: as shown in Table 5, basic information of travel service categories is stored
4. *Call information table* (call_info): it stores the historical information of users calling travel services as shown in Table 6:

#### 4.2.1. Verification of System Nonfunctional Requirements

In order to verify the accuracy and time of the prediction, it is necessary to compare the results of the reliability prediction method based on the model m_DBN before and after optimization. Since the m_DBNs model can only process continuous data before repairing, this paper simulates the use of the Circuit Services system to conduct a trip service at 200 ms intervals by continuously collecting 24 hours of data. There is also a provision that if the response time of the travel service exceeds 1000 ms, the travel service call is invalid, and the reliability of the travel service is calculated.

The reliability of the system is mainly reflected in the accuracy of service reliability prediction. Therefore, in order to optimize the reliability method based on the m_DBNs model, this paper needs to verify the accuracy of the prediction before and after, and analyze and compare the results.

This paper uses absolute mean error (MAE) and root mean square error (RMSE) to calculate the accuracy of service reliability prediction. The calculation formulas are given in the following equations:

(21) $$\begin{array}{c} \text{MAE}=\frac{\sum_{i=1}^{N}\sum_{j=1}^{L}\left|\text{PTS}\left(j\right)-\text{RTS}\left(j\right)\right|}{N\times L}, \end{array}$$

(22) $$\begin{array}{c} \text{RMSE}=\sqrt{\frac{{\sum_{i=1}^{N}\sum_{j=1}^{L}\left[\text{PTS}\left(j\right)-\text{RTS}\left(j\right)\right]}^{2}}{N\times L}}. \end{array}$$

Among them, PTS is the confidence time series obtained by prediction, RTS is the confidence time series collected in actual operation, *N* is the number of predictions, and *L* is the number of data points for the confidence of the time series. It can be seen that the smaller the amount of MAE and RMSE in the formula, the higher the accuracy of the prediction.

**Figure 5:**
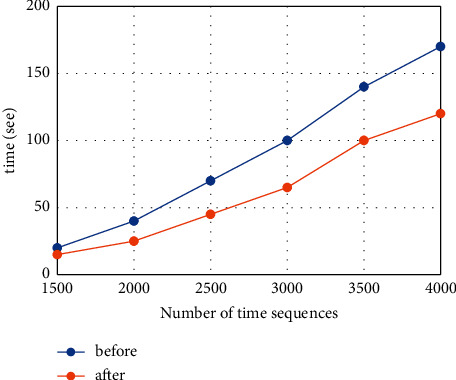
Comparison of prediction time results.

To this end, in this article, we randomly select six consecutive subdata sets from the 24-hour data collected in the simulation; calculate the time series as 1500, 2000, 2500, 3000, 3500, and 4000, respectively; and perform these subdata sets separately The reliability prediction is compared, and the prediction time is also compared. As shown in Figure 5, it is a schematic diagram of comparing the prediction time before and after the reliability method optimization based on the m_DBNs model.

The figure shows that the size of the data set has a significant impact on the reliability time. As the size of the data set increases, the time to train the conditional probability table will also increase. However, if you compare the prediction time before and after optimization of the reliability prediction method based on m_DBN, the comparison effect can be seen in the result graph. The optimized prediction time has a significantly shorter effect than before optimization. This is exactly the change in the construction of the conditional probability table.

### 4.3. The Path to Promote the Development of the Ice and Snow Sports Industry under the New Background

#### 4.3.1. Improving Industrial Integration and Strengthening the Awareness of Ice and Snow Tourism

In order to promote the high-quality development of the ice and snow tourism industry under the new situation, the integration of ice and snow tourism and related industries such as culture, sports, agriculture, education, and health should be strengthened. For example, through the joint development of the “Ice and Snow Tourism + Culture” project, to create ice and snow tourism products with different cultural connotations, creating an environmental foundation for the development of ice and snow tourism and integrate and develop a new method of “ice and snow tourism + education” to enhance the public's awareness of ice and snow sports. Therefore, as the public's awareness of ice and snow sports continues to increase, the ice and snow tourism industry has become a new growth point. “The joint development of the project will create an ice-snow hot spring health preservation and health, create an ice-snow hot spring health tourism brand, and innovate a new format of the ice and snow tourism industry. In short, it is necessary to improve the integration of the ice and snow tourism industry, strengthen the awareness of “ice and snow tourism +,” and enhance the comprehensive competitiveness and influence of the ice and snow tourism industry.

#### 4.3.2. Building a Talent Construction Platform and Improving Ice and Snow Tourism Facilities

In the process of industrial development, the government and management should vigorously support the growth of talents in the ice and snow tourism industry. On the one hand, make full use of the educational resources of colleges and universities to formulate courses and training programs related to ice and snow tourism to lay a solid foundation for the continuous provision of ice and snow professionals. At the same time, the Ice and Snow Sports Association was established to strengthen the training of experts in the ice and snow tourism industry, regularly organize professional training activities and special inspections, encourage high-quality sports professionals to actively participate in the ice and snow tourism talent training plan, and strive to create sports talents in the ice and snow tourism industry platform. To this end, the government should increase capital investment in the ice and snow tourism industry, build a talent training platform, improve the construction of local ice and snow tourism facilities, and promote the quality development of the ice and snow tourism industry.

#### 4.3.3. Grasping the Opportunity of 5G Technology and Implementing the “Ice and Snow Technology” Strategy

Seize the opportunity of 5G network development and combine local natural ice and snow tourism and existing technology to develop a new generation of culture based on ice and snow consumption and 5G high-definition tourism experience. Combine virtual reality, artificial intelligence, and other technologies to develop wearable smart devices that can be used in the fields of ice and snow sports and medical care to promote the high-quality development of information technology and the ice and snow tourism industry. Therefore, it is necessary to seize the opportunity to develop a 5G network that combines modern technologies such as ice and snow tourism with artificial intelligence, information technology, and network platforms to improve the quality of technology and services and implement them so as to further improve the “ice method and snow technology” method of ice and snow tourism to improve the development of industrial quality.

## 5. Conclusion

This article starts with the task-to-node mapping. Under several constraints such as stability and scheduling length, find the best node for each task, reduce the energy consumption of the mapping during the activity process, establish the NP problem of task mapping nodes by establishing a mathematical model, and use the optimal solution to solve it under multiple constraints, thereby transforming a node mining problem into a mathematical problem. In order to pursue the short-term reliability of data transmission in wireless sensor networks, a reliable multichannel transmission method based on optimized relay is proposed. As the leader of the tourism industry, ice and snow tourism has become an industry with the fastest growth rate, strong relevance, strong driving force, and the greatest growth potential. It has also become a new branch of economic growth.

## Figures and Tables

**Table 1:** The maximum power neighbor list of node *i* list(*i*) header.

Serial number	Channel	Distance	Transmit power	Child node collection

id,(*i*)	*c*_*j*_^*i*^	*d*(*i*,*j*)	*p*(*i*,*j*)	child(*i*)

**Table 2:** Simulation parameters.

Parameter	Value	Parameter	Value

Transmission power threshold *P*_th_ (w)	7.0 × 10^−7^	Maximum transmission radius *d*_max_ (m)	52
Channel bandwidth *B* (kHz)	15	Signal-to-noise ratio threshold *r*_th_ (dB)	6.0
Noise bandwidth *B*_*N*_ (kHz)	32	White noise *N*_0_ (*w*)	1 × 10^−7^
Data generation rate *r* (kb/s)	21	Total number of channels available	6
Maximum number of iterations *K*	200	Population size pop	55
Inertia factor *w*	0.7	EA size	20
Learning factor *c*_1_	0.7	Learning factor *c*_2_	0.3

**Table 3:** User information table.

Field	Type of data	Description

U_id	Int	User ID, primary key
u_name	Varchar(50)	Username, primary key
Pwd	Varchar(20)	Password
Email	Varchar(50)	Mailbox

**Table 4:** Tourist service information table.

Field	Type of data	Description

s_id	Int	Service ID, primary key
S_name	Varchar(50)	Service name, primary key
s_category	Int	Service category ID
Operation_name	Varchar(100)	Operation name
Wsdl	Varchar(200)	WSDL address
Input	Varchar(500)	Enter description
Output	Varchar(500)	Output description
Desc	Varchar(500)	Introduction

**Table 5:** Service classification table.

Field	Type of data	Description

Cate_id	Int	Service category *D*, primary key
Cate_name	Varchar(50)	Service category name

**Table 6:** Calling information table.

Field	Type of data	Description

u_id	Int	User ID, primary key
si_id	Int	Service ID, primary key
Start_time	Datetime	Call start time, primary key
End_time	Datetime	End time
Response_time	Int	Response time (ms)
Throughout	Double	Throughput

## Data Availability

The data used to support the findings of this study are available from the corresponding author upon request.
